# Congenital Absence of Pericardium: A Case Report and Technical Considerations in Cardiac Surgery

**DOI:** 10.7759/cureus.56885

**Published:** 2024-03-25

**Authors:** Ayush Balaji, Rishab Makam, Nabil Hussein, Mahmoud Loubani

**Affiliations:** 1 Medical Education, Hull York Medical School, York, GBR; 2 Cardiothoracic Surgery, Castle Hill Hospital, Cottingham, GBR

**Keywords:** coronary artery bypass grafting (cabg), cardiothoracic surgery, cardiopulmonary bypass, cardiac surgical procedures, pericardium, internal mammary-coronary artery anastomosis, on-pump coronary artery bypass (oncab), multivessel coronary artery disease (mvcad), congenital absence of pericardium, adult cardiac surgery

## Abstract

This case report describes a rare instance of left-sided congenital pericardial agenesis (CPA) encountered during coronary artery bypass grafting (CABG) in a 77-year-old male. In this unique case, the presence of an unusual strip of left pericardium containing the phrenic nerve posed significant surgical challenges. Special attention was required for the graft lay, ensuring adequate filling of the heart during assessment before closure, as well as emphasis on the need for generous graft length. Additionally, the evaluation of graft positioning prior to cardiopulmonary bypass was crucial. Despite these complexities, CABG was successfully performed with no complications to note. This case underscores the importance of adaptability in surgical technique to manage the unique challenges posed by CPA, leading to a positive outcome despite the atypical cardiac anatomy.

## Introduction

Congenital pericardial agenesis (CPA) is a rare cardiac anomaly, manifesting as a partial or complete absence of the pericardium. Occurring in less than 0.05% of the population, CPA is most frequently diagnosed incidentally during cardiac surgeries or during autopsy [1]. The clinical significance of CPA arises from its potential to cause complications such as cardiac herniation and strangulation, leading to ischemia, arrhythmias, and sudden cardiac death. The diagnosis of CPA, often challenging due to its asymptomatic nature and subtle radiological signs, causes significant difficulty in its diagnosis even through the utilization of various imaging modalities such as echocardiography, computed tomography (CT) scans, and MRI [2].

In the surgical context, particularly in procedures such as coronary artery bypass grafting (CABG), CPA presents unique challenges. The absence of the pericardium can significantly impact the heart's orientation and stability during heart surgery, necessitating modifications in surgical techniques and perioperative management [3,4]. Previous case reports and studies have highlighted the complexities of managing CPA and underlined the importance of intraoperative adaptability to address the atypical cardiac positioning and movement during surgery, primarily in the context of off-pump surgery, where pericardial stays are necessary to position the heart [5-7].

Our case contributes to the existing literature by providing insights into the technical considerations and strategic adaptations required for successful CABG in patients with CPA.

## Case presentation

**Figure 1:**
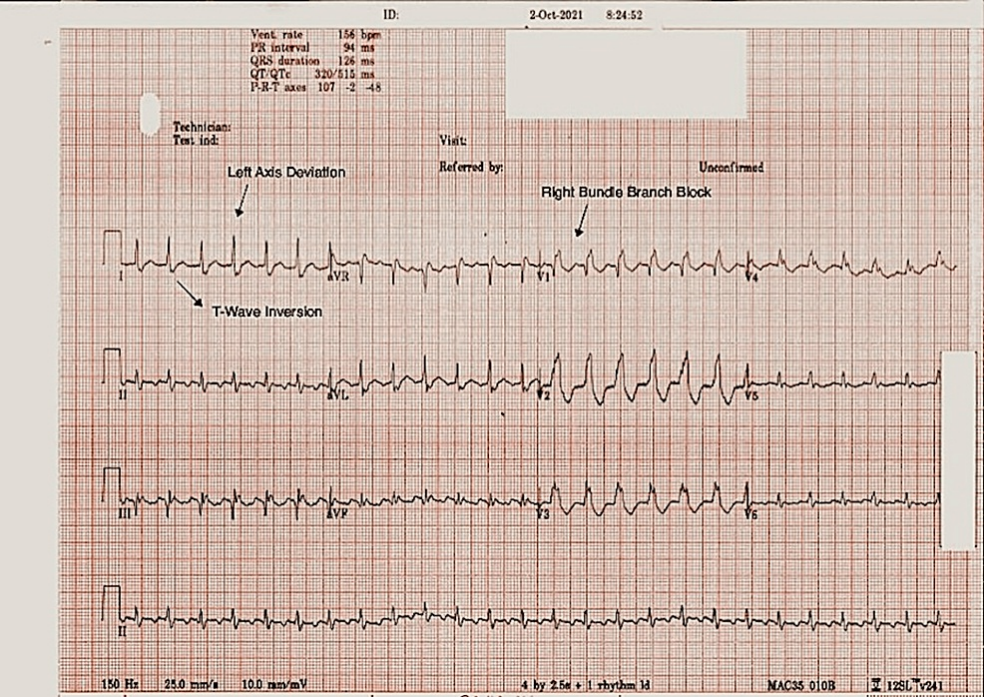
Preoperative ECG Preoperative ECG demonstrating normal sinus rhythm, left axis deviation, right bundle branch block, and T-wave inversion.

A 77-year-old male patient with a past medical history of myocardial infarction (MI), atrial fibrillation, type 2 diabetes mellitus, obesity, and prominent smoking history presented with chronic stable angina. There were no significant physical examination findings. Preoperative ECG showed sinus rhythm, left axis deviation, T-wave inversion, and right bundle branch block indicative of his past MI (Figure 1).

**Figure 2:**
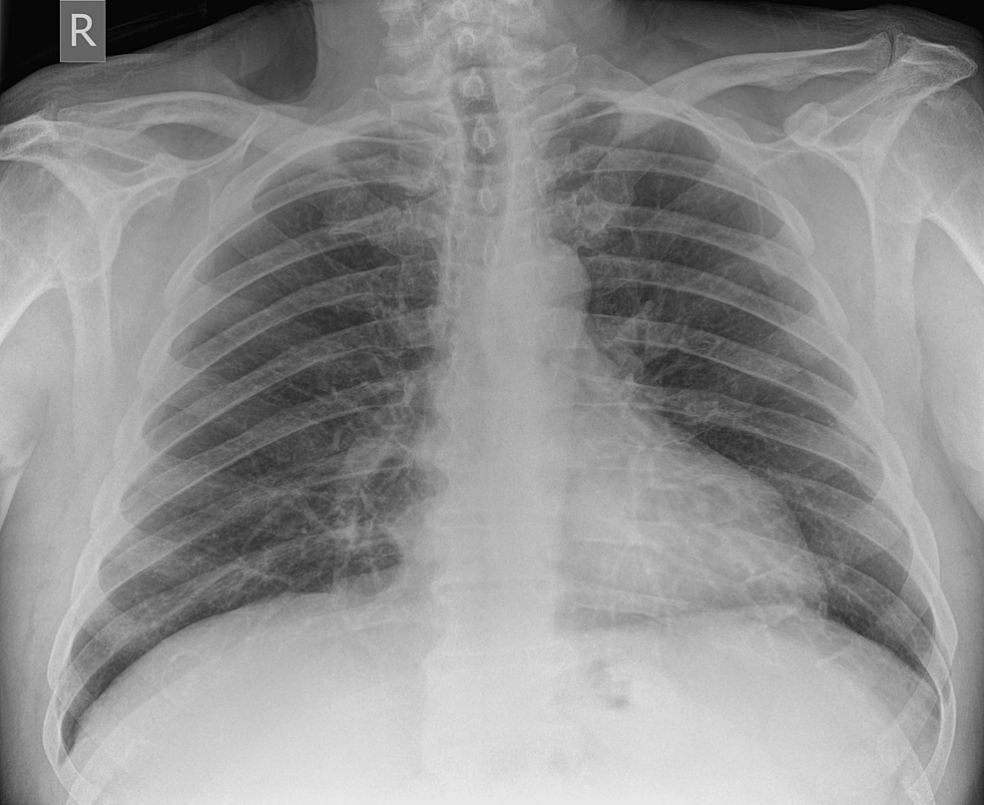
Preoperative chest X-ray Normal appearance on preoperative X-ray.

His echocardiogram was normal. Coronary angiogram showed multiple severe stenoses in the left anterior descending (LAD), circumflex (LCx), and intermediate arteries with moderate disease in the right coronary artery (RCA). The preoperative X-ray had no notable findings (Figure 2).

**Figure 3:**
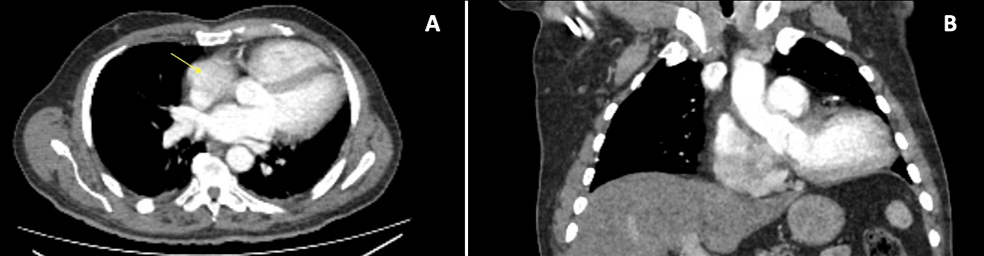
Preoperative axial (A) and coronal (B) CT images of the thorax. The position of the heart is more leftward with the right atrium in the midline.

CT of the chest showed a heart with an apex pointing to the left chest wall; however, this was not deemed significant at the time (Figure 3).

The patient was subsequently referred for surgical coronary revascularization.

Following midline sternotomy, the left internal mammary artery (LIMA) and right saphenous vein were harvested. Upon sternotomy, it was discovered that the patient had an absent left pericardium excluding a single strip suspended over the right ventricle containing the phrenic nerve and associated vessels. The strip of pericardium overlaying the midline contained a very high-positioned phrenic nerve. There was no pericardium posteriorly. The heart had herniated through the missing left pericardium into the pleural space and was rotated with a posterior-facing apex. Due to the positioning of the heart, it was difficult to access the left atrium preventing the placement of a left atrial appendage clip for the atrial fibrillation.

Following conduit harvest, cardiopulmonary bypass was achieved using two-stage right atrial and aortic cannulation. Hypothermic blood cardioplegic arrest was used, and the LAD, LCx, and intermediate arteries were assessed, which determined that the LAD and intermediate had adequate targets. This was determined from the evaluation of the coronary angiogram and on-table assessment of the caliber of the coronary arteries. The LIMA was anastomosed to the LAD and the saphenous vein graft to the intermediate with a proximal anastomosis to the aorta. The LIMA graft was laid over the strip of pericardium encapsulating the left phrenic nerve. The operation was completed in a routine fashion, and the patient was discharged on day 5 post-operatively.

## Discussion

The absence of the left pericardium poses unique anatomical challenges. The altered course of the left phrenic nerve requires careful surgical planning to prevent potential injury and subsequent complications. The absent pericardium can potentially cause difficulty in LIMA harvesting due to obstruction to view by the higher position of the phrenic nerve. The unusual rotation of the heart, with a posterior-facing apex, may further complicate the procedure [4]. However, the access to the right and left atria may be easier and assist with exposure during mitral or tricuspid valve surgery. Usually, pericardial stays allow the heart to be lifted to facilitate better positioning for coronary surgery; however, due to the CPA, the heart lays deeper in the chest cavity increasing difficulty [5].

The choice between on-pump, off-pump, and on-pump beating CABG in CPA patients is influenced by several factors [6]. On-pump surgery allows for better control and manipulation and provides optimal visualization of coronary targets as described in our case. While bowstringing effects after cross-clamp removal remain a concern, careful spatial planning can mitigate these issues [7]. Assessing coronary target positions prior to arrest and cross-clamp is imperative for envisioning the ideal graft lie. Making sure that the targets are assessed during adequate filling of the heart and being liberal with the length of grafts is important, especially with free grafts.

The decision regarding the routing of the LIMA graft, whether beneath or above the strip of residual pericardium, was pivotal. In our case, the LIMA graft was placed above the phrenic nerve (above the pericardial strip) as is routinely done. However, release of the heart into the natural position (herniating under the pericardial strip) may lead to an unnatural lay of the LIMA graft and potential kinking or stretching. To mitigate against this, a generous length of LIMA was harvested and a mid-LAD anastomosis was performed. In retrospect, the LIMA might have been better placed underneath the suspended pericardial strip to avoid any possibility of kinking or strain on the graft when placed in the natural position. Postoperatively, we observed no issues with LIMA flow, indicating positive results from the chosen approach despite the anatomical challenges.

## Conclusions

The presented case of CPA and its associated challenges during CABG highlights the complexity of this congenital anomaly and the operative flow changes it necessitates. The unique challenges posed by the absence of the left pericardium, altered cardiac anatomy, and unusual heart rotation required careful consideration and adaptation of surgical techniques.

The successful outcome of the surgery underscores the significance of graft-target evaluation, the decision-making process regarding conduit routing, graft placement, and overall perioperative management. The experience gained from this case contributes valuable insights into the technical considerations necessary when faced with CPA, providing a basis for future surgical approaches.
